# The Integration of Sub-10 nm Gate Oxide on MoS_2_ with Ultra Low Leakage and Enhanced Mobility

**DOI:** 10.1038/srep11921

**Published:** 2015-07-06

**Authors:** Wen Yang, Qing-Qing Sun, Yang Geng, Lin Chen, Peng Zhou, Shi-Jin Ding, David Wei Zhang

**Affiliations:** 1 Collaborative Innovation Center of IC Design and Manufacturing of Yangtze River Delta; 2 Institute of Advanced Nanodevices, School of Microelectronics, Fudan University, Shanghai 200433, China

## Abstract

The integration of ultra-thin gate oxide, especially at sub-10 nm region, is one of the principle problems in MoS_2_ based transistors. In this work, we demonstrate sub-10 nm uniform deposition of Al_2_O_3_ on MoS_2_ basal plane by applying ultra-low energy remote oxygen plasma pretreatment prior to atomic layer deposition. It is demonstrated that oxygen species in ultra-low energy plasma are physically adsorbed on MoS_2_ surfaces without making the flakes oxidized, and is capable of benefiting the mobility of MoS_2_ flake. Based on this method, top-gated MoS_2_ transistor with ultrathin Al_2_O_3_ dielectric is fabricated. With 6.6 nm Al_2_O_3_ as gate dielectric, the device shows gate leakage about 0.1 pA/μm^2^ at 4.5 MV/cm which is much lower than previous reports. Besides, the top-gated device shows great on/off ratio of over 10^8^, subthreshold swing (SS) of 101 mV/dec and a mobility of 28 cm^2^/Vs. With further investigations and careful optimizations, this method can play an important role in future nanoelectronics.

Transition metal dichalcogenides are a family of layered structure materials that could be used for next-generation nanoelectronic devices^1^,^2^,^3^,^4^,^5^. Stable few-layer and single-layer flakes can be obtained by the classical mechanical exfoliation which was initially used for graphene. In contrast with zero-band gap graphene, bulk MoS_2_ is a semiconductor with an indirect band gap of 1.29 eV while monolayer MoS_2_ has a direct band gap of 1.8 eV^6^,^7^. Furthermore, because of its ultrathin nature, single-layer MoS_2_ transistors are advantageous in nanometer-scale metal oxide semiconductor field-effect transistors (MOSFETs) as they are immune to short-channel effects^8^. These advantages make MoS_2_ suitable for future complementary metal oxide semiconductor (CMOS)-like logic device applications.

Excellent performance of MoS_2_ transistors adopted back gate structure have been demonstrated widely^9^,^10^,^11^. Furthermore, dual gate and top gate MoS_2_ based transistors with high-κ dielectrics have also attracted much attention recently. Since it enables the individual control of each device, the realization of high-performance dual gate or top-gated MoS_2_ transistors is a necessary step towards the practical application^1^,^8^,^12^,^13^,^14^,^15^,^16^,^17^,^18^. However, there are still some difficulties in the integration of high-κ dielectrics on MoS_2_ surfaces. Even though uniform atomic layer deposition (ALD) process of Al_2_O_3_ on MoS_2_ at 200 °C has been reported by Liu *et al.*^19^, leading to the suggestion that direct nucleation of precursors on MoS_2_ may be feasible, other works have shown that with pristine MoS_2_ flakes the direct deposition of high-κ dielectrics form island-like clusters, owing to the absence of dangling bonds on MoS_2_ basal plane^20^,^21^. It might be the use of organic pre-cleaning steps that led to uniform deposition of Al_2_O_3_ at 200 ^°^C in Liu’s work^19^. In addition, it was observed in this work that within the measurement range, neither the growth temperature nor the pulse time had an obvious impact on the topography of the Al_2_O_3_ layer grown on MoS_2_ flakes. The island-like growth of high-κ dielectrics on MoS_2_ would induce a large leakage current, therefore top-gated high-κ dielectrics in nearly all the previous reports are very thick and MoS_2_ transistors with sub-10 nm top gate dielectrics are seldom reported^1^,^8^,^12^,^13^,^14^,^15^,^16^,^17^,^18^. For example, in recent reports, the top gate dielectrics of MoS_2_ transistors are 50 nm Al_2_O_3_^16^ and 30 nm HfO_2_^18^ for the work by Pezeshki A. *et al.* and Krasnozhon D. *et al.*, respectively. Only by realizing high-quality pinhole-free and thin dielectrics over large area on MoS_2_ can the continual scaling down of MoS_2_ FETs be possible. With the shrink of dielectric thickness, especially at sub-10 nm region, the gate capacitance would be greatly improved, leading to better control of the channel and larger drive current. Some methods have been proposed to achieve uniform growth of high-κ materials on MoS_2_, such as an ultrathin metal oxide buffer layer, organic functionalization of MoS_2_ and ultraviolet-ozone exposure^20^,^21^,^22^. But most of the work just stopped at the early stage of realizing uniform growth without exploring the impacts of surface functionalization on devices performance, especially on gate leakage.

In this work, a CMOS process compatible method to achieve uniform Al_2_O_3_ growth on MoS_2_ basal plane by applying a remote O_2_ plasma treatment prior to Al_2_O_3_ growth is proposed, and top-gated MoS_2_ MOSFET with ultrathin Al_2_O_3_ dielectric deposited using this method is also studied. Notably, the Al_2_O_3_ dielectric layer is about 6.6 nm, which is the thinnest top gate dielectric ever reported, but exhibits the impressive leakage current about 0.1 pA/μm^2^ at 4.5 MV/cm. This leakage is even much smaller than that of MoS_2_ transistors capped with much thicker top gate dielectrics^1^,^8^,^13^,^16^,^17^. At the same time, the top-gated device also shows great on/off ratio of over 10^8^, subthreshold swing (SS) of 101 mV/dec and a mobility of 28 cm^2^/Vs. In addition, mechanism investigations show that after the pretreatment, oxygen atoms are physically adsorbed on the MoS_2_ surface without oxidizing it. This non-destructive physical adsorption mechanism is revealed by the advanced ultra-high-vacuum (UHV) *in-situ* analysis system. We believe it will benefit the two-dimensional electronic devices research a lot.

## Results

**Figure 1:**
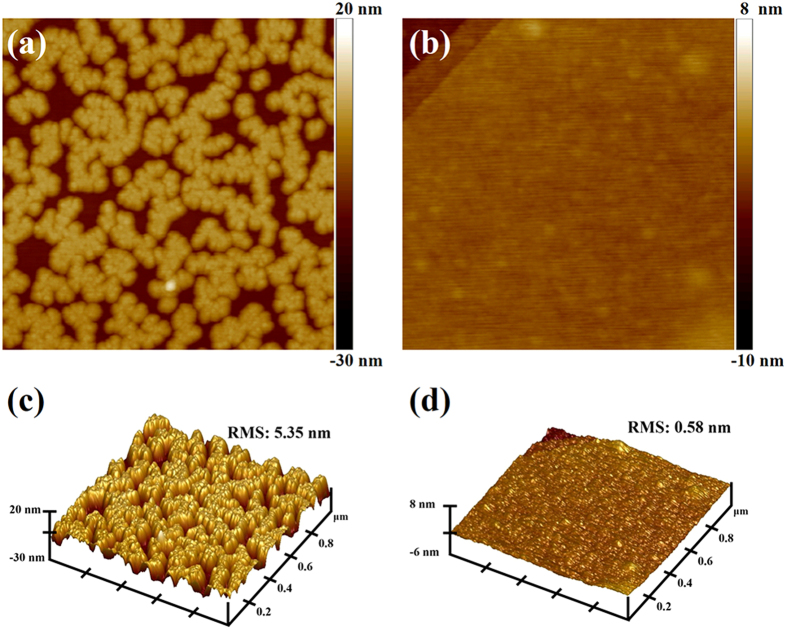
AFM images after 120 Al_2_O_3_ cycles at 200 °C. (**a**) Direct growth of the Al_2_O_3_ films on MoS_2_ flakes. (**b**) The remote O_2_ plasma pretreatment performed before ALD. (**c,d**) are the corresponding 3D images of (**a,b**), respectively. The scan size for all of the images is 1 μm by 1 μm.

Leakage current of gate oxide results in high power consumption and performance degradation of the two dimensional layered transistors^20^. To achieve uniform ALD Al_2_O_3_ growth on pristine MoS_2_, functionalization of the MoS_2_ surface is required to introduce uniform surface groups that serve as active nucleation sites for the ALD process^21^,^23^. Initially, an investigation on few-layer MoS_2_ flakes was carried out with a plasma enhanced ALD system. For the sample in Fig. 1a, 120 cycles Al_2_O_3_ was directly deposited on MoS_2_ surface at 200 °C using TMA (Trimethyl Aluminum) and H_2_O as precursors, which were kept at 18 °C in stainless bottles. By comparison, the sample in Fig. 1b was exposed to a low energy remote O_2_ plasma treatment before ALD. The pretreatment contained two steps. Each step consisted of 30 s remote O_2_ plasma exposure followed by purging with Ar for 5 s. Afterwards, 120 cycles Al_2_O_3_ was deposited in the same chamber. The different growth topography of Al_2_O_3_ on MoS_2_ basal planes are shown in Fig. 1. From Fig. 1a, it can be seen that due to the absence of dangling bonds on MoS_2_ basal plane, direct deposition of Al_2_O_3_ films are in forms of island-like clusters with large pinholes, and the lateral size of most pinholes are over 100 nm. In this situation, it is easy to imagine that the gate dielectric of top gate MoS_2_ transistors have to be thick to form uniform film which is necessary to keep the leakage current at a sufficient lower level. By contrast, with remote oxygen plasma treatment prior to ALD, the grown Al_2_O_3_ film is completely uniform on MoS_2_ surface as shown in Fig. 1b. The difference is even more evident by the comparison between Fig. 1c,d, which are the corresponding AFM 3D images of Fig. 1a,b. Root mean square (RMS) of the ~12 nm directly deposited film is 5.35 nm, and it decreases to only 0.58 nm with remote oxygen plasma pretreatment, which is about 10% of the value in Fig. 1c. It is obvious that the remote O_2_ plasma pretreatment served as an effective method to supply sufficient nucleation sites to achieve a uniform ALD process. More details about the direct deposition of Al_2_O_3_ on pristine MoS_2_ basal planes are available in the Supplementary Information. According to previous reports with graphene^24^ and MoS_2_^25^,^26^, heavy exposure to an oxygen plasma (typically a direct plasma) completely etches the flakes and results in the substitution of sulfur with oxygen and re-deposition of the surface materials during etching of the MoS_2_ flakes. Therefore, further analysis is needed to investigate the impact of the low energy remote oxygen plasma.

**Figure 2:**
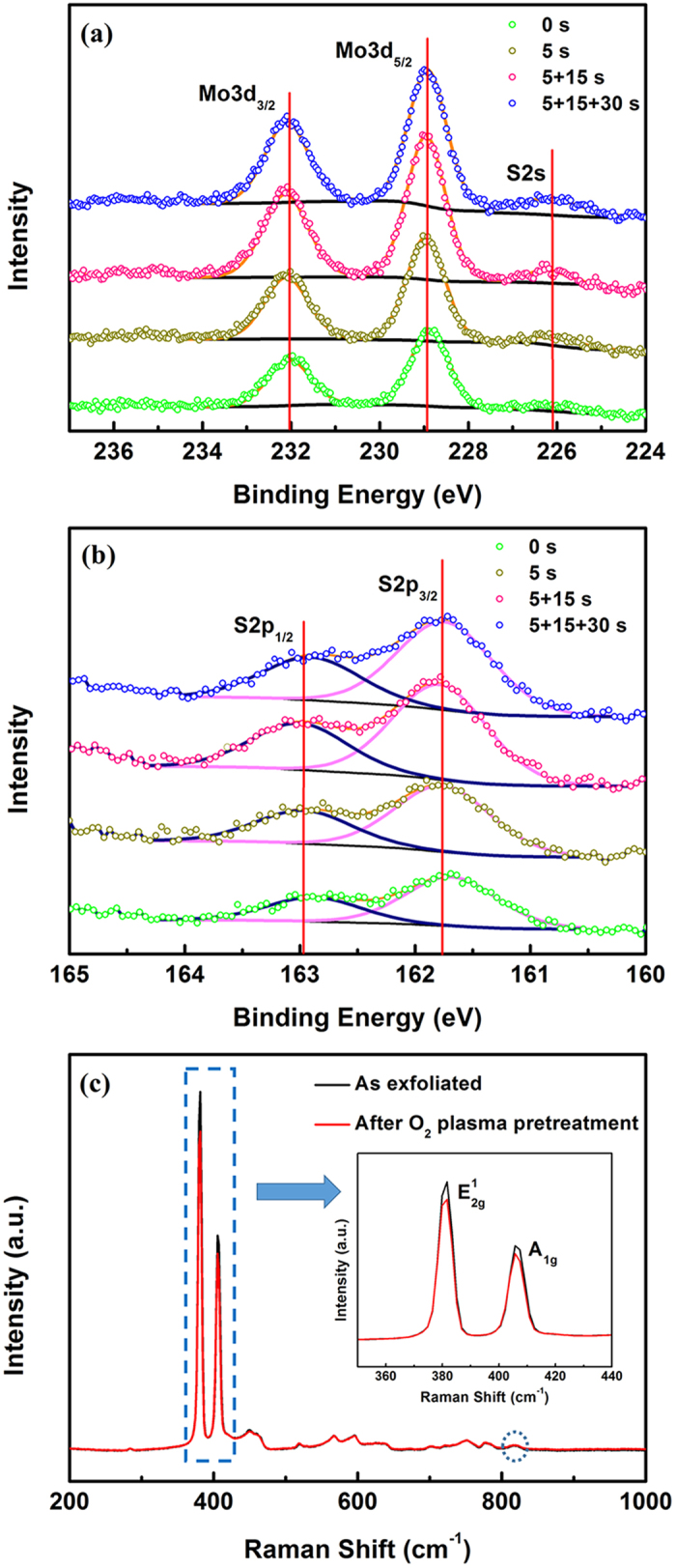
(**a**) *In-situ* XPS spectra showing the Mo 3d and S 2s core level peak regions before and after remote O_2_ plasma treatments for various time. (**b**) *In-situ* XPS spectra showing the S 2p core level peak regions. (**c**) Raman spectra before and after the remote O_2_ plasma treatments. The inset is a zoom of the spectra showing the 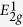 and A_1g_ peaks in MoS_2_. The blue dotted circle in (**c**) is used to highlight the peak near 820 cm^−1^ which is used to estimate the extent of MoS_2_ oxidation.

*In-situ* investigations were performed to gain an insight into the mechanisms of uniform growth after a remote O_2_ plasma pretreatment, looking at whether the MoS_2_ flakes were oxidized during the treatment or oxygen atoms were adsorbed on the MoS_2_ surface and acted as nucleation sites in the following ALD process. In the *in-situ* cluster system, the ALD system was connected to an X-ray photoelectron spectroscopy (XPS) system through a high-vacuum transfer line (the pressure was about 10^−10^ mbar). The sample was first transferred from the load-lock chamber to the XPS chamber for characterization. It was then transferred to the ALD chamber for 5 s remote O_2_ plasma treatment. Afterwards, the sample was transferred back into the XPS chamber for further measurements. This procedure was repeated twice with treatment time of 15 and 30 s. The Mo 3d, S 2s and S 2p regions of the XPS spectra are shown in Fig. 2a,b, respectively. The Mo 3d spectra consists of peaks around 229 and 232 eV, corresponding to the Mo^4+^ 3d_5/2_ and Mo^4+^ 3d_3/2_ components, respectively. Similar peaks appear around 161.8 and 163 eV, referring to the S 2p_3/2_ and S 2p_1/2_ components of the S 2p region, respectively. In Fig. 2, all these peaks have nearly no shift after the remote O_2_ plasma treatment, implying that the chemical bonds were not damaged during the process. Also, no peaks appear around 236 eV, demonstrating that the molybdenum atoms were not oxidized after the plasma treatment^27^,^28^. This is different to the previous results where a direct RF-oxygen plasma was applied^25^,^26^,^28^. Based on the discussion above, it seems that remote oxygen plasma treatment is a surface-based process. When a remote oxygen plasma was applied, oxygen atoms were adsorbed onto the MoS_2_ surfaces and acted as nucleation sites for the ALD process. The remote plasma was gentle enough such that the flakes were not oxidized during the treatments. When the flakes were transferred into the XPS chamber through the transfer line, the adsorbed oxygen atoms desorbed, caused by the high vacuum in the transfer line and the reductive environment due to the working principle of the molecular pump. In addition, it should be noticed that the intensity of both Mo 3d and S 2p peaks varied with the measurement position actually. As there were a lot of MoS_2_ flakes on the tested SiO_2_ substrate, and tiny position shifts between adjacent measurements were inevitable, results obtained from different XPS measurements may contain information from different MoS_2_ flakes. Meanwhile, since the thickness and density of MoS_2_ flakes varied according to their locations, there were some intensity differences in both Mo 3d and S 2p components with treatment time. In this case, the intensity of Mo 3d and S 2p components with 5 + 15 s remote oxygen plasma treatment happened to be the maximum.

To further verify the results obtained by XPS, Raman spectra of the sample before and after the oxygen plasma treatments mentioned above were obtained in air using a 514-nm laser (Fig. 2c). The inset of Fig. 2c displays the spectra enlarged, showing the 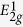 and A_1g_ modes for MoS_2_ at ~380 and 405 cm^−1^. According to the work of Bertrand P. A.^29^, the in-plane 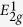 mode is brought about by the opposite vibration of two S atoms with respect to a Mo atom and the A_1g_ mode is generated from the out-of-plane vibration of S atoms in opposite directions. From the inset of Fig. 2c, consistency of peak positions between these two spectra at the 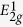 and A_1g_ modes can be observed before and after the sample undergoing the remote oxygen plasma treatments, implying that bonding situations of Mo and S atoms didn’t change. In addition, the peak that centers near 820 cm^−1^ could be used to estimate the extent of oxidation that occurred^30^. In case that the MoS_2_ flakes were oxidized, this peak would be more defined and intense after the treatments. As expected, this peak showed no intensity difference before and after the remote O_2_ plasma pretreatments, indicating that the MoS_2_ flakes remained un-oxidized during the pretreatments. From these results, it is clear that during the treatments, the remote O_2_ plasma is gentle enough to avoid damaging the MoS_2_ flakes. Instead, the produced oxygen species are adsorbed onto the MoS_2_ surface and serve as nucleation sites for the initial TMA pulses during the ALD process.

**Figure 3:**
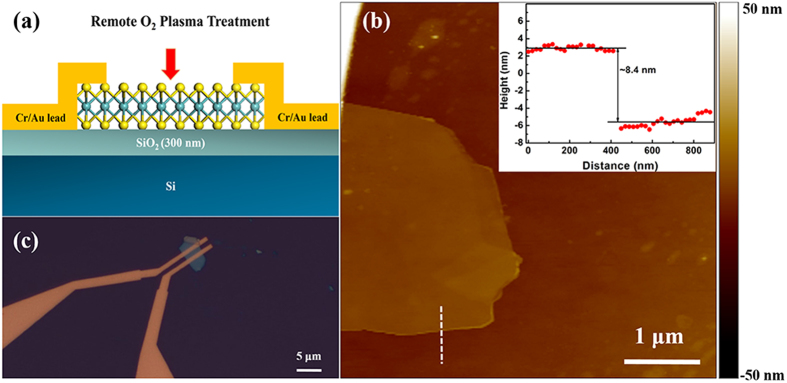
Fabrication of few-layer MoS_2_ transistors. (**a**) Cross-sectional schematic of a back-gated MoS_2_ transistor treated with remote O_2_ plasma. (**b**) AFM image of a few- layer MoS_2_ flake on a 300-nm SiO_2_ substrate. The inset shows the cross-sectional plot along the white line in (**b**). (**c**) Optical image of the device using the flake in (**b**) as the channel. The substrate acts as a back gate. The channel length and width of the device are 1 and 4.2 μm, respectively.

**Figure 4:**
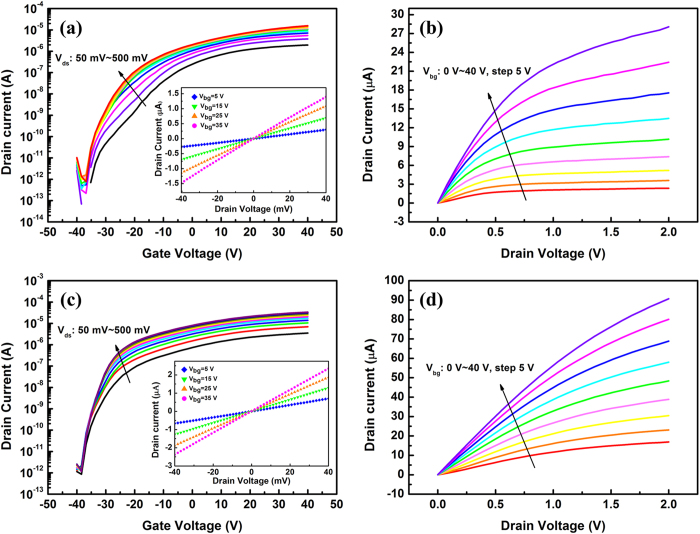
Electrical characterization of the few-layer MoS_2_ transistor. (**a,b**) are the transfer and output characteristics for the original MoS_2_ transistor. (**c,d**) are the transfer and output curves for the same device after a 60 s remote O_2_ plasma treatment. The insets in (**a**) and (**c**) show the I_ds_–V_ds_ curves with back-gate voltages of 5, 15, 25 and 35 V. The linear relationship between the current and voltage within −40 ~ 40 mV indicated that the Cr/Au electrodes form perfect ohmic contacts. All these curves were acquired at room temperature.

As the mobility is of great significance when evaluating the performance of electronic devices, back-gated MoS_2_-based field effect transistors were fabricated to estimate the impact of the remote O_2_ plasma pretreatment on the device mobility. A cross-sectional schematic of the MoS_2_ transistor with the remote O_2_ plasma treatment is shown in Fig. 3a, and thickness of the MoS_2_ flake is about 8.4 nm as shown in Fig. 3b, which correspond to ~12 monolayers. The corresponding photograph of the device structure is shown in Fig. 3c. For the electrical characterization, one of the electrodes acts as a drain and the other one is grounded, acting as a source. Initially, Cr/Au electrodes are used with a MoS_2_ channel by applying a source-drain bias (V_ds_) to the pair of electrodes as shown in Fig. 3c and a gate bias (V_bg_) to the heavily doped silicon substrate. As shown in the insets in Fig. 4a,c, the I_ds_ – V_ds_ curves are all linear in the range from −40 to 40 mV with or without the remote O_2_ plasma pretreatment, indicating that the Cr/Au contacts are ohmic contacts. The transfer and output characteristics for the MoS_2_ transistor before and after 60 s remote O_2_ plasma pretreatment are obtained for comparison. The data presented in Fig. 4 show typical n-type transistor behavior with an on/off ratio (I_on_/I_off_) over 10^7^. This high on/off ratio compared to graphene transistors is attributed to the large band gap of MoS_2_. It is also observed in Fig. 4a,c that both the shape of the transfer curves and the values of the ON current are improved after 60 s remote O_2_ plasma pretreatment. For example, the transfer current at V_ds_ = 500 mV increases from 1.56 × 10^−5^ to 3.38 × 10^−5^ A after a 60 s remote O_2_ plasma pretreatment. A low field-effect mobility is extracted using the Equation:[1]
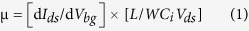
where L = 1 μm is the channel length, W = 4.2 μm is the channel width, and C_i_ = 1.15 × 10^−8^ F/cm^2^ is the capacitance density between the channel and the back gate (details of the mobility extraction can be found in Supplementary Information). Results show that the mobility increases from the original value of 22.15 to 33.57 cm^2^/Vs after 60 s remote O_2_ plasma pretreatment. Moreover, from the comparison between Fig. 4b (without pretreatment) and Fig. 4d (with pretreatment), the output current increase greatly as well after 60 s remote O_2_ plasma pretreatment. Taking the I_ds_ – V_ds_ curve under V_bg_ = 40 V for example, the output current increases from 2.81 × 10^−5^ to 9.07 × 10^−5^ A after 60 s remote O_2_ plasma pretreatment. Furthermore, for both the transfer and the output characteristics, excellent field-effect behavior is observed.

**Figure 5:**
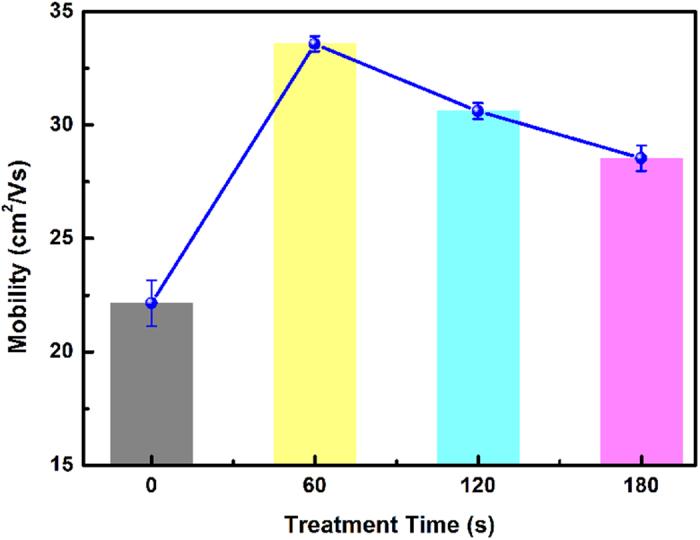
Device mobility evolution with the remote O_2_ plasma pretreatment time.

The evolution of the device mobility is tested with different pretreatment time. As shown in Fig. 5, the mobility of the device reaches its peak value with 60 s pretreatment, and decreases slightly with a prolonged pretreatment time, but still higher than the original value. Error bars of Fig. 5 are contributed from repeated measurements each time. To verify the experimental phenomenon, many other back-gate devices were fabricated and tested in the same manner. As expected, a similar phenomenon was observed, proving these results were not a coincidence. This indicates that there is a compromise between the mobility and pretreatment time. It should be noticed that the optimized pretreatment time should be different with different instruments.

## Discussion

**Figure 6:**
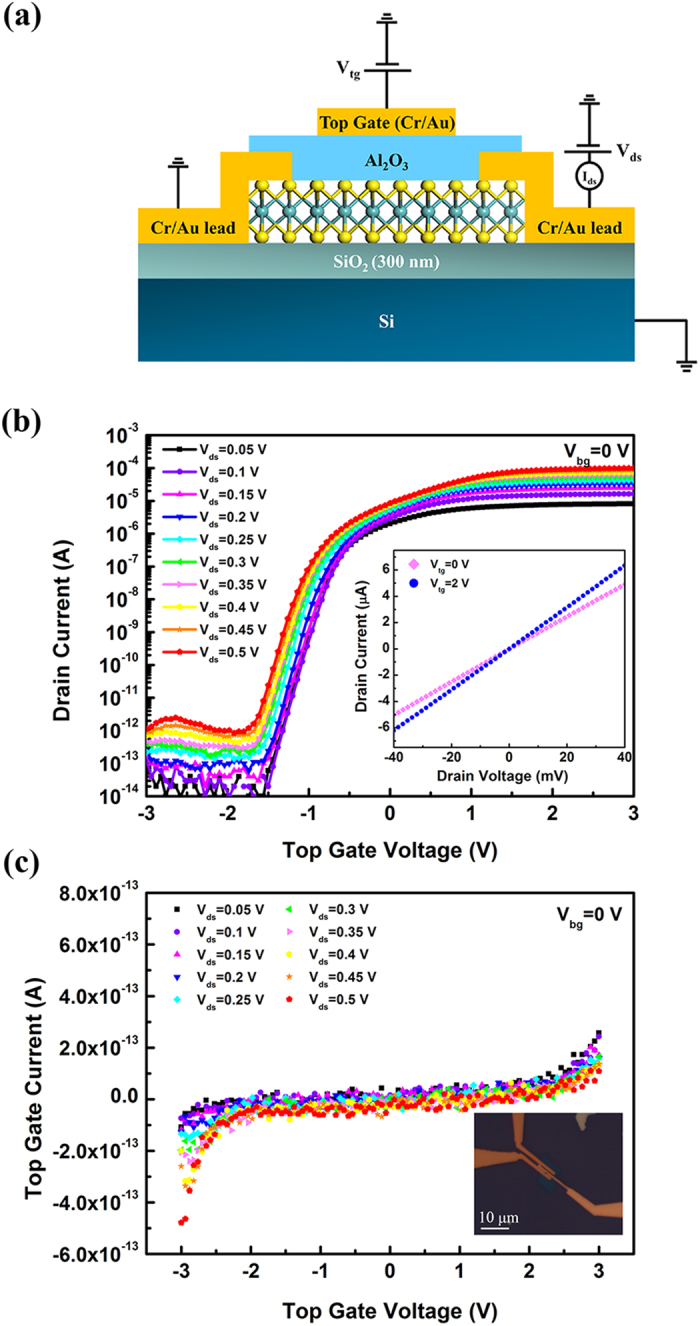
Top gate characteristics of a few-layer MoS_2_ transistor. (**a**) Cross-sectional schematic of the top-gated devices together with the electrical connections. (**b**) I_ds_ – V_tg_ curves with V_ds_ ranging from 50 mV to 500 mV. The inset shows the I_ds_ – V_ds_ curves with the top gate voltages of 0 V and 2 V. (**c**) Top gate leakage current of the device. Optical image of the top gate device is attached as the inset of (**c**). Top gate dielectric of this device is 60 cycles Al_2_O_3_ deposited with 60 s remote oxygen plasma pretreatment. All these measurements were performed at room temperature with the back gate grounded.

Figure 6 shows the top gate transfer characteristics and leakage current of a few layer MoS_2_ transistor with top gate dielectric deposited using remote oxygen plasma pretreatment. Top gate dielectric of this device is 60 cycles Al_2_O_3_ (about 6.6 nm) which was deposited at 300 °C with 60 s remote oxygen plasma pretreatment. L = 1 μm and W = 5 μm are the channel length and channel width, respectively. For all the measurements in Fig. 6, back gate of the device is grounded as shown in Fig. 6a. From the inset of Fig. 6b, the linear relationship between I_ds_ and V_ds_ within −40 mV −40 mV indicates that Cr/Au electrodes form perfect ohmic contacts with the MoS_2_ channel. In addition, for all the transfer curves presented in Fig. 6b, great on/off ratio of the current over 10^8^ can be observed within the ±3 V range of the top gate voltage. Top gate leakage current is also measured in the same device. Compared to previously reported top-gate leakage of 2 pA/μm^2^ within 2 MV/cm^1^,^8^, as shown in Fig. 6c, the leakage current is less than 5 × 10^−13^ A (about 0.1 pA/μm^2^) in the measurement range of −3 V to 3 V (4.5 MV/cm). This leakage is much smaller and at same time with an ultrathin gate oxide. The field effect mobility of this top gate device is extracted using Equation (1) discussed above, which was 28 cm^2^/Vs under V_ds_ = 0.5 V with the SS to be 101 mV/dec.

In summary, uniform Al_2_O_3_ growth on the MoS_2_ basal plane was successfully achieved by applying a remote O_2_ plasma pretreatment before ALD, and the mechanism was investigated systematically. After a remote oxygen plasma pretreatment, the oxygen species are physically adsorbed onto the surfaces of the MoS_2_ flakes and act as nucleation sites for the ALD cycles. The transport studies reveal an extra benefit of this method, which is that unlike many other methods that might sacrifice the device mobility to achieve uniform high-κ growth, this method improves the device mobility by 50%. Furthermore, top-gated MoS_2_ transistor with ultrathin Al_2_O_3_ dielectric was also fabricated. With only 6.6 nm Al_2_O_3_ as dielectric, which is the thinnest top gate dielectric ever reported so far, the device shows impressive leakage about 0.1 pA/μm^2^ at 4.5 MV/cm. Besides, the top-gated device shows great on/off ratio of over 10^8^, subthreshold swing (SS) of 101 mV/dec and a mobility of 28 cm^2^/Vs. According to the mechanism, it is believed that this method can also be adopted for high-κ growth on other two dimensional nanostructures and used in other devices. With further investigations and optimizations, this method could play an important role in the future nanoelectronics.

## Methods

### Preparation of the few-layer MoS_2_ flakes

Ultrathin layers of MoS_2_ were obtained from bulk crystals (SPI supplies Brand) using the classical tape-based mechanical exfoliation method commonly used for graphene, then transferred onto degenerately doped Si substrates covered with 300 nm SiO_2_. The thicknesses of these flakes were determined with a Bruker Multimode 8 atomic force microscope (AFM).

### Atomic layer deposition of Al_2_O_3_ on MoS_2_ flakes and characterization

Some of the MoS_2_ flakes were loaded into the Picosun R200 ALD chamber for direct Al_2_O_3_ deposition. During the deposition, TMA and H_2_O served as the aluminum and oxygen precursors, respectively, and different growth temperatures and pulse time were adopted to observe their impacts. For some of the flakes, the remote O_2_ plasma pretreatments were carried out in the same chamber before Al_2_O_3_ was deposited. Here, “remote” means that the plasma source is located remotely from the substrate stage, such that the substrate is not involved in the generation of the plasma. It is then carried to the sample surface by the carrier gas^31^. The X-ray photoelectron spectroscopy (XPS) system used was made by SPECS GmbH. The X-ray source for data acquisition during the *in-situ* characterization was SPECS XR50 X-ray source. Considering that the signal intensity was not so strong due to the low density of MoS_2_ flakes on the substrate and the signal intensity might be further weakened by using an X-ray monochromator, we finally carried out the *in-situ* characterization using a non-monochromatic XPS source. In the *in-situ* XPS measurements, all the spectra were taken using a Mg Kα X-ray source (hν = 1253.6 eV). The working pressure in the ultra-high-vacuum (UHV) chamber for the data acquisition was maintained at the magnitude of 10^−10^ mbar. The element library and the quantification factors used during measurements were provided via the system SpecsLab2 software, and after the measurements, the data analysis was carried out using CasaXPS software. The binding energies in the XPS spectra were calibrated in the conventional way against the adventitious carbon C 1s singlet (E_b_ = 284.6 eV). The Raman spectra of the MoS_2_ flakes before the remote O_2_ plasma treatment were measured with a Renishaw inVia Raman microscope in air using a 514 nm laser. Then after going through the *in-situ* XPS characterization mentioned above, Raman spectra of the same sample was measured again with all the measurement settings to be the same.

### MoS_2_ transistors fabrication

The degenerately doped Si substrate and the 300-nm SiO_2_ layer served as the back gate and the gate dielectric, respectively. The source and drain contacts were formed using electron-beam lithography followed by deposition of 10 nm Cr and 70 nm Au. The electrical properties of the transistors were measured with an Agilent B1500 semiconductor device parameter analyzer.

## Additional Information

**How to cite this article**: Yang, W. *et al.* The Integration of Sub-10 nm Gate Oxide on MoS_2_ with Ultra Low Leakage and Enhanced Mobility. *Sci. Rep.* **5**, 11921; doi: 10.1038/srep11921 (2015).

## Supplementary Material

Supplementary Information
