# Supplementary material for: The Integration of Sub-10 nm Gate Oxide on MoS2 with Ultra Low Leakage and Enhanced Mobility
Source: Sci Rep. 2015 Jul 6;5:11921. doi: 10.1038/srep11921 (PMC4491716; doi:10.1038/srep11921)
Supplement: Supplementary Information [file srep11921-s1.doc]

**Supplementary Information for:**

**The Integration of Sub-10 nm gate oxide on MoS_2_ with Ultra Low Leakage and Enhanced Mobility**

Wen Yang^1,2^, Qing-Qing Sun^1,2^*, Yang Geng^1,2^, Lin Chen^1,2^, Peng Zhou^1,2^, Shi-Jin Ding^1,2^ and David Wei Zhang^1,2^*

^1^Collaborative Innovation Center of IC Design and Manufacturing of Yangtze River Delta

^2^Institute of Advanced Nanodevices, School of Microelectronics, Fudan University, Shanghai 200433, China

* Corresponding author:

Email address: qqsun@fudan.edu.cn, dwzhang@fudan.edu.cn

**Direct deposition of Al_2_O_3_ on pristine MoS_2_ flake:**

For pristine graphene, the failure of direct ALD of high-κ materials on the basal plane has been reported and the growth of a high-κ materials have only been observed at the graphene edges and at defect sites^1^. Since there are no dangling bonds or surface groups on graphene’s basal plane, it is difficult for precursors to nucleate on graphene surfaces. In contrast with graphene, discontinuous growth on the MoS_2_ basal plane was observed, as shown in Figure S1. Within the temperature range from 120 °C to 400 °C, the direct deposition of Al_2_O_3_ on MoS_2_ basal planes form island-like clusters, showing weak temperature dependence. This is completely different from the previous reports where a strong dependence on the growth temperature and uniform deposition under 200 °C has been reported^2,3^. Recently, the non-uniformity phenomenon has also been reported by Mcdonnell et al. with HfO_2_ deposition^4^. In addition, when the pulse time during the ALD process is sufficient, prolonging the pulse time does not lead to the adsorption of extra precursors on the MoS_2_ surface (Figure S2).

**Back-gated device mobility evolution after remote O_2_ plasma pretreatment:**

For mechanism verification, several devices were fabricated to test the evolution of the mobility with time in air after a remote O_2_ plasma pretreatment. In Figure S3a, no high-κ deposition was performed after the pretreatment. The results show that the mobility significantly increased after the pretreatment. When the devices were placed in air, the value quickly dropped initially, then the speed decreased. After 6 days, the mobility had almost decreased to its original value before the pretreatment, remaining almost unchanged. It can be understood that after the pretreatment, the oxygen species first physically adsorbed onto the MoS_2_ surfaces, acting as a screening layer, which suppressed the Coulomb scattering in the MoS_2_ flakes to some extent, leading to an increase in the mobility. When the devices were placed in air, the adsorbed oxygen atoms desorbed quickly at first, and then with fewer and fewer species on the surfaces, this desorption speed gradually slowed down. Finally, when nearly all the oxygen species had desorbed, the mobility returned to its original value and remained nearly unchanged.

The device mobility evolution with Al_2_O_3_ coverage is shown in Figure S3b for comparison. The values at the zero point on the time axis referred to the device mobility immediately after 100 cycles Al_2_O_3_ deposition. The Al_2_O_3_ film was deposited at 300 °C with 60 s remote O_2_ plasma pretreatment. As shown in Figure S3b, after Al_2_O_3_ coverage, the device mobility increased immediately from 13.77 to 30.27 cm^2^/Vs, then lightly decreased to a relatively stable state after 3 days. Besides, different from Figure S3a in which the devices mobility decreased to the original values, after the sample placed in air for a whole week, mobility of the device with Al_2_O_3_ coverage still remained stable at a much higher level than the original value.

**Field-effect mobility extraction:**

To illustrate in details how the field-effect mobility were extracted, the transfer curve of the back-gated device in Figure 3 before remote oxygen plasma treatment is taken for an example. As shown in Figure S4, firstly the transfer curve under V_ds_=100 mV was measured at room temperature, and the corresponding transconductance
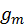
 was calculated by the first order differential of the curve. Then the field-effect mobility was extracted using the equation:


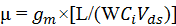


In the equation,
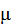
 is the target field-effect electron mobility. L=1 μm and W=4.2 μm are the channel length and channel width, respectively.
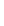

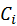
=1.15×10^−8^ F/cm^2^ is the capacitance density between the channel and the back gate (
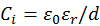
,
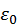
 is the vacuum permittivity,
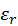
=3.9 is the dielectric constant of SiO_2_, and *d*=300 nm is SiO_2_ thickness). V_ds_=0.1 V is the drain voltage applied, and
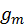
=1.036×10^−7^ S is the peak value of the transconductance curve. The limited condition for the application of this equation is that V_ds_ should be small enough, which was definitely satisfied. As a result, based on the discussions above, the extracted field-effect mobility was 21.44 cm^2^/Vs.

**Comparison with related reports:**

Table S1 has summarized the performance of the top-gated transistors in related reports. For comparison, electrical performance of the top-gated transistor in this work is added in Table S1 as No.12. It can be seen that with our dielectric being the thinnest in all of the work, top gate leakage of our device is the smallest ever reported to the best of our knowledge. This important result shows that the top gate dielectric of our device is of high quality and the remote oxygen plasma pretreatment is an effective method for uniform high-k deposition on MoS_2_. Moreover, taking the channel thickness into consideration, the comprehensive performance of our device is at leading level for few-layer top-gated MoS_2_ transistors in Table S1.

**Table S1.** Summary of Top-gated MoS_2_ transistors performance in related reports

| **NO.** | **Publication Year** | **Journal** | **Top gate dielectric** | **Top gate mobility at room temperature (cm^2^/Vs)** | **SS (mV/dec)** | **Leakage**  **(pA/um^2^)** |
| --- | --- | --- | --- | --- | --- | --- |
| **1**^[5]^ | 2014 | Nano lett. | 30 nm HfO_2_ | Monolayer:85  Trilayer: 51 | \ | \ |
| **2**^[6]^ | 2014 | Adv. Mater. | Y_2_O_3_ buffer layer+ 28 nm HfO_2_ | Few layer: 63.7 | over 100 | Larger than 1 @ 4.5 MV/cm |
|  |  |  | Y_2_O_3_ buffer layer+ 9 nm HfO_2_ | \ | 65-74 | \ |
| **3**^[7]^ | 2014 | J. Mater. Chem. C | 50 nm Al_2_O_3_ | Trilayer: 5 | 229 | about 0.2 @ 2 MV/cm |
| **4**^[8]^ | 2014 | Solid State Electron. | 15 nm Al_2_O_3_ | Monolayer: 6.2 | 780 | \ |
| **5**^[9]^ | 2013 | Nat. Mater. | 30 nm HfO_2_ | Monolayer: smaller than 63 | \ | \ |
| **6**^[10]^ | 2013 | Nano lett. | 17.5 nm ZrO_2_ | Trilayer: 25 | \ | \ |
| **7**^[11]^ | 2013 | Nano lett. | Al seeding layer + 15 nm Al_2_O_3_ | Monolayer: 21.6 | \ | \ |
| **8**^[12]^ | 2013 | Nanoscale | 40 nm Al_2_O_3_ | Monolayer: 170  Bilayer: 25  Trilayer: 15 | Monolayer: 90  Bilayer: 500  Trilayer: 1100 | \ |
| **9**^[3]^ | 2012 | Electr. Device Lett. | 16 nm Al_2_O_3_ | 23 layers: 4.13 | 140 | 2 @ 2 MV/cm |
| **10**^[13]^ | 2012 | Nano lett. | 50 nm Al_2_O_3_ | Monolayer: 80  Bilayer: 27  Trilayer: 10 | Larger than 300 | about 0.2 @ 1.4 MV/cm |
| **11**^[14]^ | 2011 | Nat. Nanotechnol. | 30 nm HfO_2_ | \ | Monolayer: 74 | 2@1 MV/cm |
| **12*** | **\** | **\** | **6.6 nm Al_2_O_3_** | **Few layer: 28** | **101** | **0.1 @ 4.5 MV/cm** |

* No.12 refers to the electrical performance of top-gated transistor in this work.


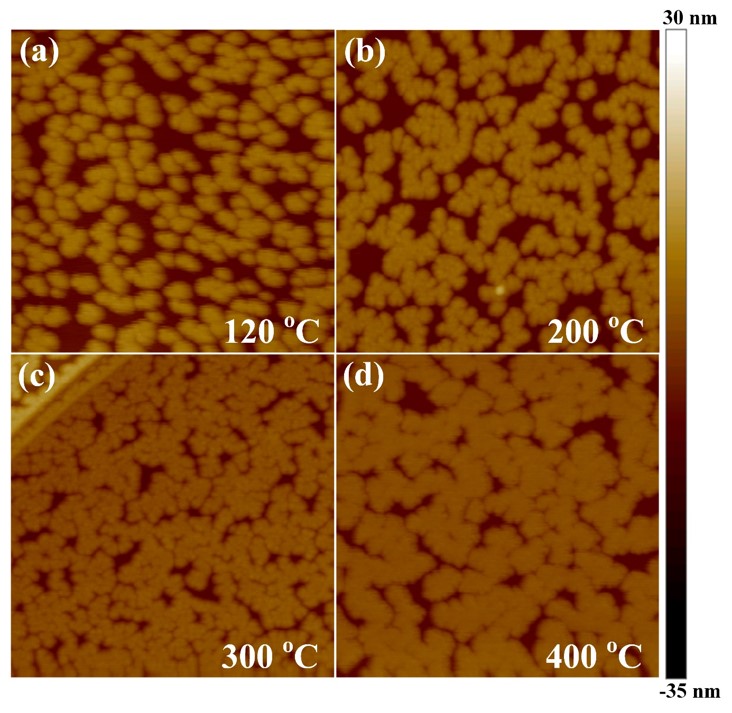


**Figure S1.** Atomic force microscope images of the MoS_2_ surfaces after 120 ALD cycles of Al_2_O_3_ at 120 °C, 200 °C, 300 °C and 400 °C. For the different samples, the pulse and purge time of precursors were all the same during deposition. All the images were taken in a 1 μm by 1 μm area.


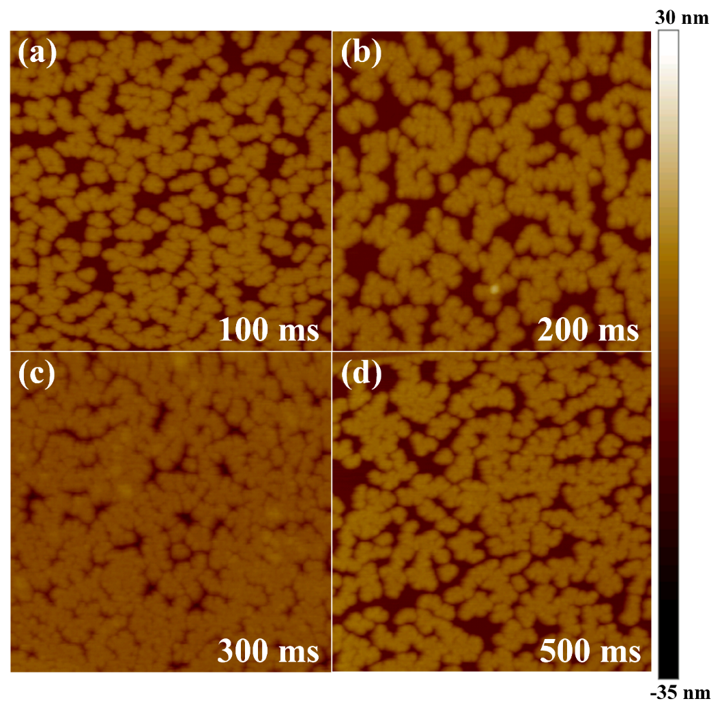


**Figure S2.** AFM images of the MoS_2_ surfaces after 120 ALD cycles of Al_2_O_3_ with different pulse time for the TMA and H_2_O precursors. The deposition temperature was set to 200 °C. All the images were taken over a 1 μm by 1 μm area. When the pulse time of the TMA and H_2_O were set to 100 ms, this was sufficient to achieve uniform Al_2_O_3_ growth on the substrates with dangling bonds such as Si and SiO_2_.


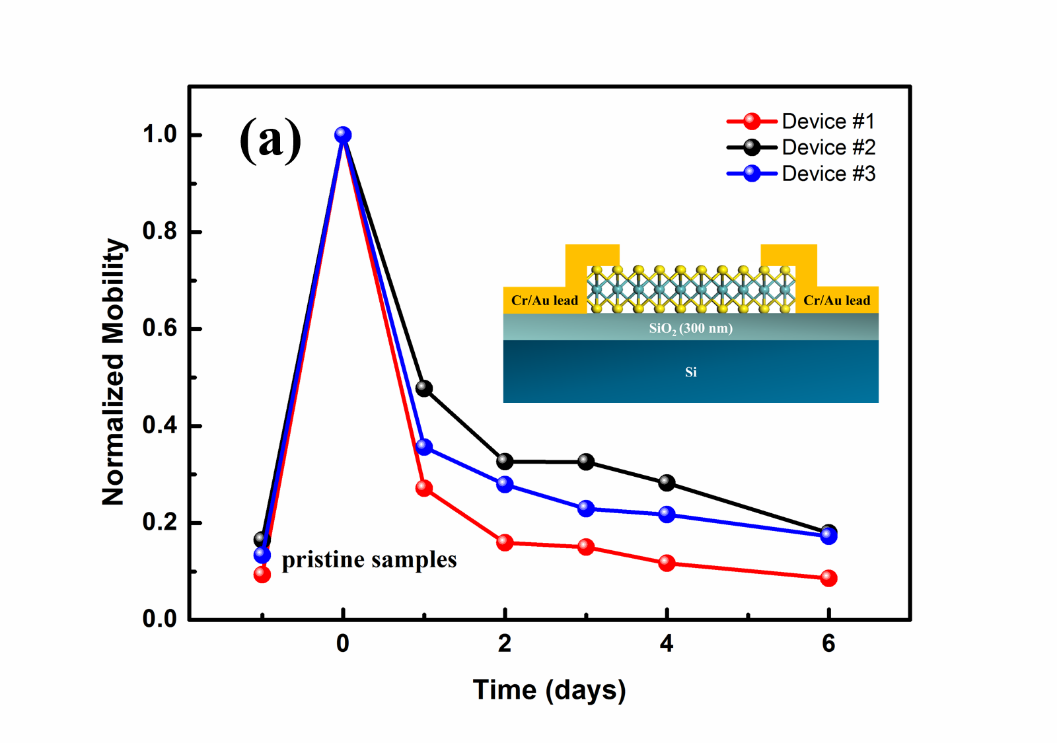


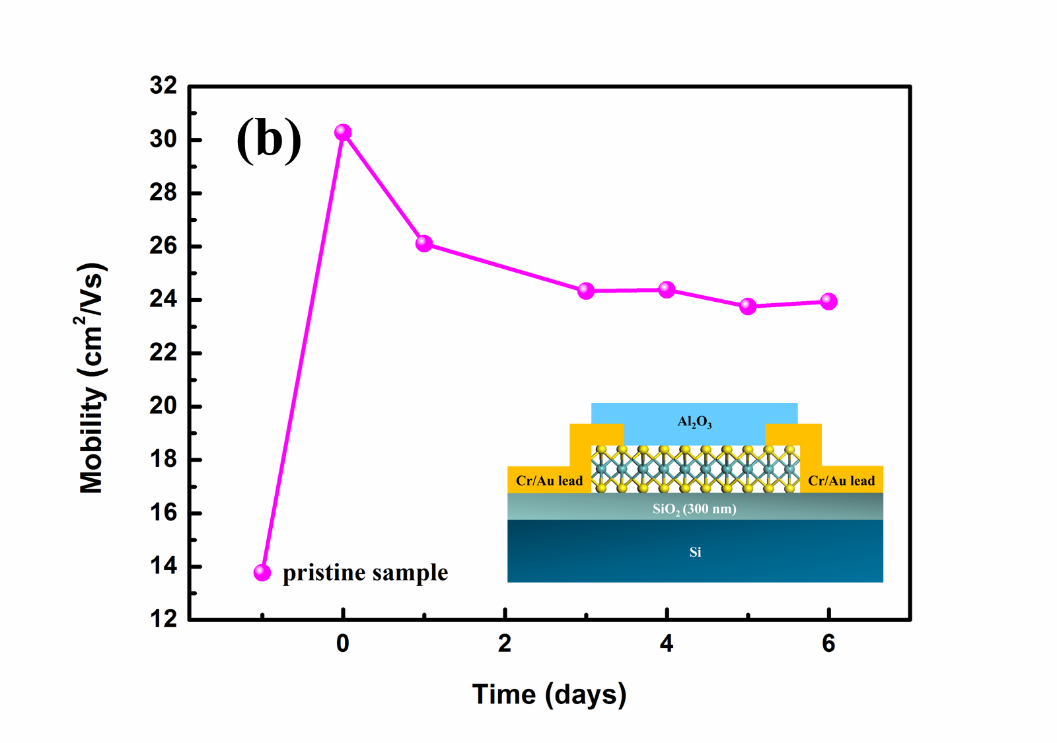


**Figure S3.** Field-effect mobility evolution. (a) Evolution of the normalized device mobility with time in air after 60 s remote O_2_ plasma pretreatment. (b) Evolution of device mobility with time in air after 100 ALD cycles of Al_2_O_3_ deposited with 60 s remote oxygen plasma pretreatment. Insets of both figures are schematics of the tested devices. The values at the zero point on the time axis corresponded to the mobility immediately after remote plasma pretreatment or Al_2_O_3_ deposition. Mobility in both of the figures were extracted at V_ds_=100 mV.


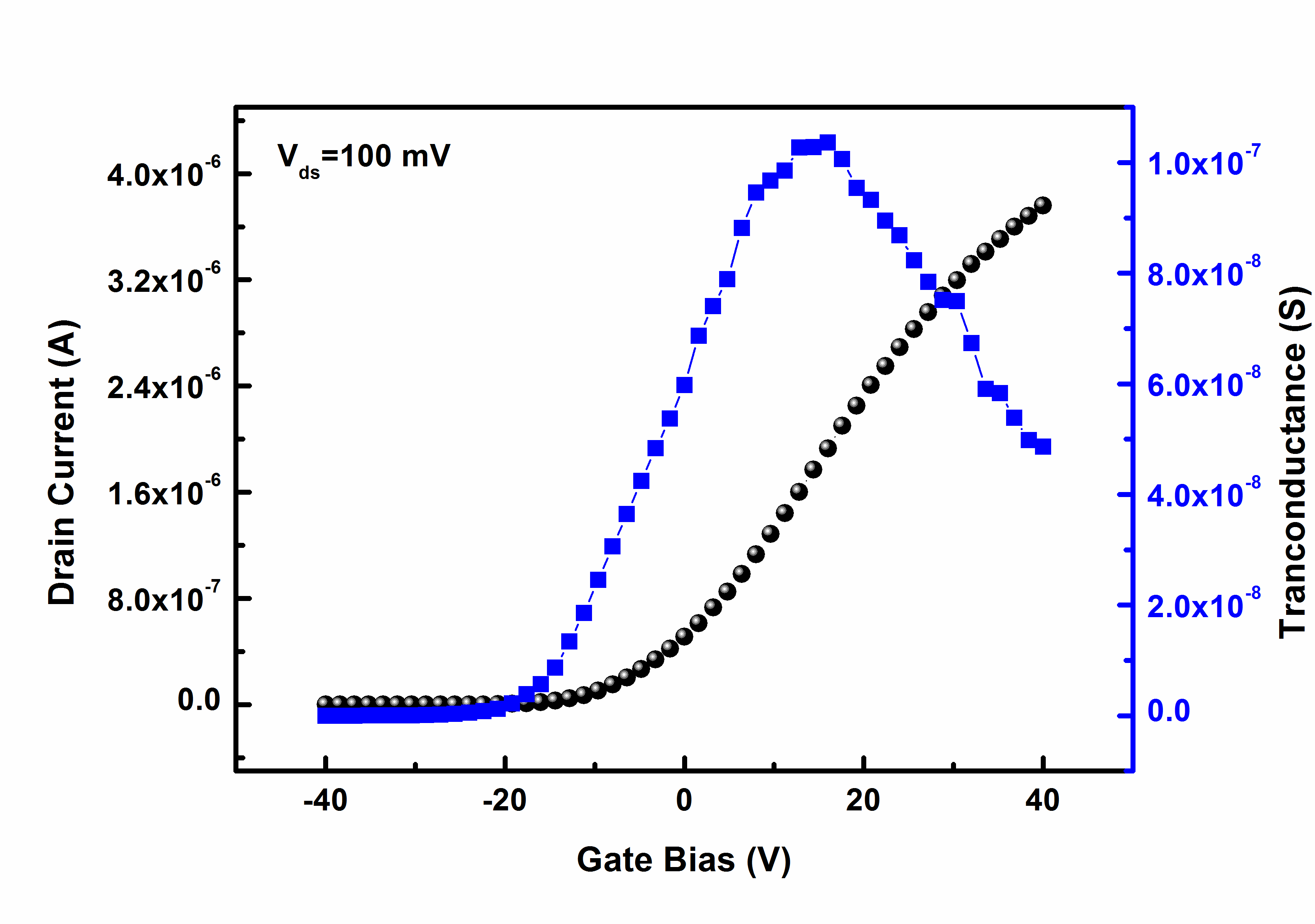


**Figure S4.** I_ds_-V_bg_ curve and the corresponding transconductance of the back-gated device in Figure 3. The curves were measured before the remote oxygen plasma treatment. The dotted line with black solid balls is the transfer curve, and the dotted line with blue squares refers to the corresponding transconductance.

**References**

1. Wang, X.R., Tabakman, S.M. & Dai, H.J. Atomic layer deposition of metal oxides on pristine and functionalized graphene. *J. Am. Chem. Soc.* **130**, 8152-8153 (2008).
2. Liu, H., Xu, K., Zhang, X. & Ye, P.D. The integration of high-k dielectric on two-dimensional crystals by atomic layer deposition. Appl. Phys. Lett. 100, 152115 (2012).
3. Liu, H. & Ye, P. MoS_2_ Dual-Gate MOSFET with Atomic-Layer-Deposited Al_2_O_3_ as Top-Gate Dielectric. *IEEE Electron Dev. Lett.* **33**, 546-548 (2012).
4. McDonnell, S. *et al*. HfO_2_ on MoS_2_ by Atomic Layer Deposition: Adsorption Mechanisms and Thickness Scalability. *ACS Nano* **7**, 10354-10361 (2013).
5. Krasnozhon, D., Lembke, D., Nyffeler, C., Leblebici, Y. & Kis, A. MoS_2_ Transistors Operating at Gigahertz Frequencies. *Nano Lett.* **14**, 5905-5911 (2014).
6. Zou, X. *et al*. Interface Engineering for High-Performance Top-Gated MoS_2_ Field-Effect Transistors. *Adv. Mater.* **26**, 6255-6261 (2014).
7. Pezeshki, A. *et al*. Top and back gate molybdenum disulfide transistors coupled for logic and photo-inverter operation. *J. Mater. Chem. C* **2**, 8023-8028 (2014).
8. Shah, P.B. *et al*. Analysis of temperature dependent hysteresis in MoS_2_ field effect transistors for high frequency applications. *Solid-State Electron.* **91**, 87-90 (2014).
9. Radisavljevic, B. & Kis, A. Mobility engineering and a metal–insulator transition in monolayer MoS_2_. *Nat. Mater.* **12**, 815-820 (2013).
10. Fang, H. *et al*. Degenerate n-Doping of Few-Layer Transition Metal Dichalcogenides by Potassium. *Nano Lett.* **13**, 1991-1995 (2013).
11. Liu, H. *et al*. Statistical Study of Deep Submicron Dual-Gated Field-Effect Transistors on Monolayer Chemical Vapor Deposition Molybdenum Disulfide Films. *Nano Lett.* **13**, 2640-2646 (2013).
12. Min, S. *et al*. Nanosheet thickness-modulated MoS_2_ dielectric property evidenced by field-effect transistor performance. *Nanoscale* **5**, 548-551 (2013).
13. Lee, H.S. *et al*. MoS_2_ Nanosheet Phototransistors with Thickness-Modulated Optical Energy Gap. *Nano Lett.* **12**, 3695-3700 (2012).
14. Radisavljevic, B., Radenovic, A., Brivio, J., Giacometti, V. & Kis, A. Single-layer MoS_2_ transistors. *Nat. Nanotechnol.* **6**, 147-150 (2011).
